# Development of Molecular Markers and a Preliminary Selection Index for Yield-Related QTLs in *Secale cereale* subsp. *segetale*

**DOI:** 10.3390/genes17101179

**Published:** 2026-09-24

**Authors:** Juan Liu, Yunjie Yang, Zhenbo Zhai, Xiaoyi Liu, Ying Liu, Jiarui Zou, Yanping Yang, Yonghe Che, Lai Wei

**Affiliations:** 1 Hebei Key Laboratory of Crop Stress Biology, Qinhuangdao 066600, China; 15660971377@163.com (J.L.); yangyunjieren@163.com (Y.Y.); 15090484869@126.com (Z.Z.); 18740983260@163.com (X.L.); l17796724330@163.com (Y.L.); kellen0204@163.com (J.Z.); c2163589106@163.com (Y.Y.); 2 College of Agronomy and Biotechnology, Hebei Normal University of Science and Technology, Qinhuangdao 066600, China

**Keywords:** *S. cereale* subsp. *segetale*, yield-related traits, SLAF sequencing, molecular markers, marker-assisted selection index

## Abstract

As a close relative of common wheat, rye is a forage crop of significant research value in China. **Background**: A weedy rye, *Secale cereale* subsp. *segetale* found in Xinjiang China, is characterized by superior yield-related traits such as grain length, grain width, spike length, spike width, plant height, and tiller number. The identification of genes controlling these traits provides significant assistance for rye breeding. However, its characteristics of cross-pollination and self-incompatibility complicate the genetic analysis of these traits. Despite extensive studies on stress tolerance in rye, the genetic basis of yield-related traits has been the subject of only a few studies. **Methods**: Our previous research preliminarily identified quantitative trait loci (QTLs) affecting yield-related traits through specific-locus amplified-fragment sequencing (SLAF-seq). In this study, we focused on the targeted development of molecular detection markers by identifying single nucleotide polymorphisms (SNPs) or simple sequence repeats (SSRs) tightly linked to these yield-related QTLs. Linked SNP/SSR markers were developed and screened across seven diverse rye populations (C_2_, A_3_–A_7_). **Results**: Through screening and validation in diverse rye populations, two SSR markers and seven KASP (Kompetitive Allele Specific PCR) markers capable of distinguishing phenotypic traits were identified. These markers effectively genotyped tiller number, stem diameter, and spikelet number. Subsequently, by annotating and screening genes adjacent to the target QTLs, we identified multiple candidate genes, including *ScWN2R01G507400.1*, *ScWN4R01G301500.1*, *ScWN4R01G335200.1*, and *ScWN4R01G352500.1*. These genes primarily encode proteins such as those containing valine-glutamine (VQ) motifs, F-Box proteins, MATH-BTB domain-containing proteins. By integrating QTL phenotypic variation explained (PVE) from SLAF-seq, molecular markers, and post-harvest agronomic traits, we constructed an exploratory marker-assisted selection index for preliminary evaluation of rye germplasm, and suggest that independent validation in larger populations and multiple environments is required. **Conclusions**: These findings provide valuable references for investigating the genetic basis of yield related traits in rye.

## 1. Introduction

Rye (*Secale cereale* L.), a diploid plant of the Poaceae family with 14 chromosomes, is a member of the Triticeae subtribe and a closely related species of wheat (*Triticum aestivum* L.). It is cultivated worldwide, primarily in Asia and Europe. A weedy rye, Xingjiang weedy rye (*S. cereale* subsp. *segetale*) found in Xinjiang of China is annual, cross-pollinated rye [1]. This subspecies survives catastrophic frost and exhibits vigorous spring resurgence, locally referred to as ‘Bingmai’ [2]. Researchers not only accomplished the genome assembly and analysis of Weining rye (a Chinese cultivated rye) but also obtained a chromosome-scale genome assembly of Lo7 rye as well as an early whole-genome draft sequence of Lo7 rye, providing substantial resources for studies on rye and related cereal crops [3,4,5].

Yield, as one of the primary objectives in Poaceae crop breeding, is controlled by multiple agronomic traits, such as spike length, spike width, spikelet number, grain length, grain width, tiller number, and thousand-grain weight [6,7,8]. These traits are mostly quantitative traits and controlled by multiple genes. To date, numerous genes controlling these traits have been identified in Poaceae crops. For instance, multiple QTLs associated with grain weight and grain shape in wheat have been discovered on various chromosomes except 1D, 4B, and 4D [9,10,11,12]. Specifically, a QTL controlling thousand-grain weight and grain width was mapped to chromosome 4A (100.56–103.39 Mb), with *TraesCS4A03G0191200* encoding a WD40 protein proposed as a candidate gene [13]. On chromosome 2DS, a 3.97 Mb physical region linked to grain length and thousand-grain weight was identified, with *TraesCS2D03G0923000* proposed as a candidate gene [14]. Additionally, a QTL associated with grain weight was detected on chromosome 2D within a 10.7 Mb physical interval near Ppd-D1, and TaDA1, a negative regulator of kernel size, was located within 5 Mb of this region [15].

Gene-sequencing technology, also referred to as DNA-sequencing technology, is a rapidly developing technique in recent years that obtains the base sequence of target DNA fragments; its emergence has played an important role in the development of life sciences and medicine research [16]. SLAF-seq is a reduced-representation genome-sequencing method based on second-generation sequencing. It enables high-throughput development and genotyping of molecular markers (such as SNPs, InDels, and SSRs), and is widely used in the mapping of QTLs in crops [17]. For instance, the approach is applied in maize (*Zea mays*) for the genetic mapping of QTLs controlling resistance to corn leaf aphid, enabling the identification of candidate genes involved in jasmonic acid signaling [18]. Concurrently, SLAF-seq technology enables the rapid and cost-effective development of a large number of SNP markers, facilitating the construction of a high-density genetic map. For instance, researchers applied this method to sequence a recombinant inbred line population in soybean (*Glycine max*), developed a high-density genetic map covering 2387.6 cM, and successfully mapped multiple QTLs associated with hundred-seed weight, thereby revealing the polygenic architecture underlying this important yield trait [19].

Advances in DNA-sequencing technologies have promoted the widespread application of sequencing-based molecular markers in genetics and breeding. Among these, SNP-based markers (including SNP itself and its derivative technologies like KASP) represent the dominant category of sequencing-derived markers. KASP is a competitive allele-specific PCR technology developed by LGC (Laboratory of the Government Chemist). As a type of SNP marker, it employs universal fluorescent probes to accurately perform biallelic genotyping of specific polymorphic sites in genomic DNA [20]. KASP has been widely used in plant breeding. For instance, in wheat, a KASP marker targeting *TaAHL67* was developed and validated for identifying superior haplotypes associated with increased thousand-grain weight [21]; in fiber-type hemp, a panel of seven core KASP markers was developed to construct unique DNA fingerprints, enabling efficient cultivar discrimination and germplasm management [22]; and in wheat drought tolerance research, two KASP markers, *TaDreb-B1* and *1-FEH w3*, were utilized to efficiently select drought-tolerant plants [23].

As a close relative of wheat, rye (*S. cereale* L.) is an important food and forage crop carrying valuable genes related to disease resistance, stress tolerance, and yield improvement in its R genome. Investigating yield-related traits (such as spike length, grain size, and tillering capacity) in rye is of great significance for developing high-yielding wheat and rye varieties, as well as improving forage quality. Due to self-incompatibility, conventional breeding approaches are less efficient in rye genetic improvement, and this reproductive barrier also poses a core obstacle to genetic research. To overcome these limitations, CP populations (cross-pollinated populations) are commonly used; a CP population is an F_1_ segregating population generated by the double pseudo-testcross between two highly heterozygous parents. According to Grattapaglia and Sederoff [24], this configuration allows single-dose markers from either parent to segregate in a 1:1 testcross ratio, thereby enabling simultaneous recovery of recombination information from both parental meioses. Because its marker segregation mimics a testcross, it is particularly suitable for SNP-based linkage map construction and QTL analysis in self-incompatible cross-pollinated species such as rye. Against this background, the development of a marker-assisted selection index represents a practical effort to facilitate selection in rye breeding. Therefore, our research group previously utilized SLAF-seq technology to construct a high-density genetic map from a cross-pollinated population between cultivated rye (*S. cereale* L.) and wild rye (*S. cereale* subsp. *segetale*), and performed preliminary mapping of yield-related QTLs [25]. Based on these QTL regions, this study developed molecular markers that can be used for selecting rye germplasm with superior yield traits and for identifying candidate genes influencing yield performance. In wheat-rye translocation lines, chromosome 4R has been implicated as a potential carrier of favorable alleles for yield and resistance traits, suggesting that this chromosome may be of special importance for rye genetic improvement. These findings provide a foundation for breeding elite rye varieties through marker-assisted selection and functional gene utilization.

## 2. Materials and Methods

### 2.1. Plant Materials

The rye materials used in this study were provided by Hebei Key Laboratory of Crop Stress Biology. Field cultivation was conducted at the university’s Agronomy Experimental Station. The experimental rye populations were sown with row spacing of 30 cm and plant spacing of 15 cm, followed by routine standard agronomic practices. The designations and nomenclature of the experimental and validation populations are listed in Table 1. Population C_1_ (158 individuals), planted in the autumn of 2018, was used for the material for SLAF-seq analysis and genetic-map construction [25]. The C_2_ population was planted in the autumn of 2020, a total of 376 individual plants were grown by hole sowing under the same spacing. Individual plants were used as the experimental units for phenotypic and genotypic analyses. The A_3_–A_7_ populations (12 plants each), planted in the autumn of 2020 using the same planting method as C_2_, along with C_2_, were utilized as marker validation populations. Populations C_1_ and C_2_ represent a cross-pollinated (CP) hybrid population, whereas A_3_–A_7_ comprise open-pollinated populations from a self-incompatible variety. At the maturity, the following yield-related traits were evaluated: tiller number (TN), available tiller number (ATN), ear of grain-stem node length (ESL), stem diameter (SD), biological yield (BY), spike length (SL), spike width (SWd), root length (RL), spikelet number per spike (SNS), kernel number per spike (KNS), kernel weight per spike (KWS), spike weight (SW), plant height (PH), plant yield (PY), grain length (GL), grain width (GW) and thousand-grain weight (TGW) [26].

### 2.2. Development of SSR Molecular Markers

Based on the yield-related QTLs previously identified by our group, SSR sequences were identified using the SSR Hunter v1.3 software [27]. Primers were designed based on the obtained SSR sequences with the Primer Premier 5.0, and their specificity was verified using TBtools v2.468 [28]. These candidate SSR markers were subsequently tested on plants from the C_1_ population. The PCR reaction mixture and amplification program (Table S1) were used. Primers that successfully amplified products were selected and further validated for polymorphism across diverse genetic backgrounds using plants from populations A_3_–A_7_ and a subset of C_2_. Those primers that exhibited consistent amplification were subsequently used to genotype the entire C_2_ population. The resulting genotyping data, combined with agronomic trait data, were subjected to *t*-tests using the SPSS software (version 26.0) to identify molecular markers significantly associated with the corresponding traits.

### 2.3. Development of KASP Molecular Markers

For QTL segments where SSR marker design was unsuccessful, KASP markers were developed based on tightly linked SNP loci. Primers were designed using the Poly Marker web tool with the Weining rye genome [4,5] as the reference. Primer specificity was verified using TBtools v2.468 [28]. Each KASP assay comprises two allele-specific forward primers (labeled with FAM and HEX fluorescent dyes, respectively) and one common reverse primer. The KASP assays were initially tested on a subset of plants from the C_1_ population. PCR amplification and fluorescence signal detection were performed using the CFX Connect Real-Time PCR Detection System (Bio-Rad Laboratories, Inc., Hercules, CA, USA). The reaction conditions are detailed in Table S2.

After adding all experimental components into a sterile 96-well plate, the plate was briefly centrifuged to mix the contents and collect them at the bottom of the wells. The plate was then placed in the thermocycler for amplification. To prevent fluorescence signal interference, all steps involving fluorescent dyes were performed under low-light conditions. The thermocycling program for KASP assays is shown in Table S2.

Following amplification, fluorescence signals were captured by the instrument’s detection system. Assays yielding clear cluster separation in genotyping plots were selected. These selected KASP assays were then validated on a subset of plants from populations C_2_ and A_3_–A_7_ to confirm genotyping stability across different genetic backgrounds. Assays demonstrating stable genotyping were used to genotype all plants in the C_2_ population. The genotyping data were integrated with the phenotypic trait data, and associations were evaluated using *t*-tests in the SPSS software (version 26.0).

### 2.4. Candidate Genes Analysis and Screening

The genome sequence of Weining rye [3] was used as a reference to pinpoint the physical locations of QTLs for which molecular markers had been successfully developed and validated. Genes located in the vicinity of these QTL intervals were then extracted. Their coding sequences (CDSs) were retrieved using TBtools v2.468 software and functional annotation was performed by eggNOG-mapper [29]. Transcriptomic expression data for these genes in Weining rye [3] were obtained from the WheatOmics database [30]. Expression patterns were visualized as a heatmap using TBtools v2.468. Candidate genes putatively involved in plant growth and development were evaluated based on functional analysis of orthologs in related Poaceae crops.

### 2.5. Collinearity Analysis

Collinearity analysis between rye and the wheat A subgenome (*Triticum urartu*) was performed using MCScanX [31] with default parameters. The identified syntenic blocks between the two species were visualized using the Dual Synteny Plot program in TBtools v2.468 [28].

### 2.6. Development of an Exploratory Marker-Assisted Selection Index

An exploratory marker-assisted selection index (Z-function) was constructed by integrating phenotypic trait data, molecular marker genotypes, QTL information (including physical positions and PVE values) from the single-environment C_1_ SLAF-seq analysis [25], and parental phenotypic data.

## 3. Results

### 3.1. SLAF-Seq Analysis and QTL Identification

In our previous study [25], genotyping of the C_1_ population (158 individuals) and its parents using SLAF-seq technology identified 57 candidate QTLs and 293 linked SNP markers. After merging closely linked markers within 1-LOD support intervals, a total of 28 QTL clusters were retained for 17 agronomic traits. These QTLs were systematically named using the convention ‘qTrait-chromosome’ (with suffixes ‘a’, ‘b’, ‘c’ for multiple clusters on the same chromosome). Their detailed distribution across chromosomes is summarized in Table 1. Details of the candidate QTLs for yield-related traits in rye are provided in Table S3.

### 3.2. Development and Screening of SSR Markers

Based on the 57 identified QTLs (which were later consolidated into 28 clusters for presentation in Table 2), we selected closely linked SSR sequences and designed corresponding primers. A total of 62 SSR markers, designated SSR-X (where X is a serial number), were developed. These markers were first screened by PCR amplification and electrophoresis in the C_1_ population (158 individuals), resulting in 12 markers that produced clear and typable target bands. The polymorphic markers were further validated for stability and genotyping reliability across diverse genetic backgrounds using the biparental population C_2_ and the five natural populations A_3_–A_7_. Ultimately, eight SSR markers showed stable amplification and consistent genotyping across all tested populations (Table 3).

These eight selected SSR markers were then used to genotype all plants in the large biparental population C_2_. Integration of genotypic data with phenotypic measurements revealed statistically significant associations (*p* < 0.05) for markers SSR18 and SSR26, as determined by *t*-tests comparing the phenotypic values between plants carrying Allele 1 and those carrying Allele 2, with heterozygotes excluded. These associations should be regarded as preliminary marker–trait evidence. The results of the association analysis and representative electrophoretograms are shown in Table 4 and Figure 1, respectively.

### 3.3. Development and Screening of KASP Markers

For QTL regions where polymorphic SSR markers could not be developed, we designed 44 KASP markers based on linked SNP sites. These were designated KM-X (X denotes a serial number). These markers were screened for polymorphism in the C_1_ population, and 15 KASP assays were identified as suitable for robust genotyping.

Subsequent validation across the C_2_ and A_3_–A_7_ populations confirmed that all 15 KASP assays produced stable and clear genotyping clusters (Table 5). These validated assays were then used to genotype all 376 plants in the C_2_ population. Among these markers, both KM33 and KM36 were associated with SNS. The associations between KASP markers and traits, as determined by *t*-tests comparing the phenotypic values between plants carrying Allele 1 and those carrying Allele 2 (with heterozygotes excluded), are summarized in Table 6. These results were not corrected for multiple testing. Representative genotyping plots are shown in Figure 2.

### 3.4. Screening of Candidate Genes

For the QTL regions identified in this study, we identified 80 candidate genes located within or near these intervals. Transcriptomic data of Weining rye [3], including expression levels of candidate genes under normal growth conditions, were obtained from the WheatOmics database. The data encompassed multiple tissues: roots, stems, leaves, and spikes at the heading stage; flowers at 5, 10, and 15 days after flowering (DAF); and grains at 10, 20, 30, and 40 DAF. After filtering out non-expressed genes, thirty-five candidates remained and were functionally annotated (Table 7). Their expression patterns across different tissues were visualized as a heatmap using TBtools v2.468 (Figure 3).

Based on known functions of yield-related genes in Poaceae, we further identified nine high-potential candidate genes from the annotated list. These encode proteins such as VQ motifs, F-Box proteins, UDP-glucosyltransferases (UGTs), zinc finger proteins, and MATH-BTB domain-containing proteins. These proteins are known to be involved in diverse molecular pathways regulating plant growth and development. It should be noted that these candidates were selected solely on the basis of positional information and expression evidence from related species. Their actual roles in rye-yield traits remain to be experimentally confirmed. These nine genes are, therefore, high-priority targets for future functional validation studies on yield traits in rye.

### 3.5. Construction of an Exploratory Marker-Assisted Selection Index

We constructed an exploratory marker-assisted selection index (Equation (1)) by integrating QTL PVE values derived from the single-environment C_1_ SLAF-seq analysis, molecular marker genotypes, and post-harvest agronomic trait data. The PVE values were used directly as coefficients for the corresponding QTL-associated traits. The detailed derivation and calculation steps are provided in Table S4. Compared to a conventional membership function model D = Σ [Wi × F(Xi)], where F(Xi) = (Xi − Xmin)/(Xmax − Xmin), based solely on phenotypic principal component analysis, our genomic model more effectively identifies superior rye germplasm. It achieves this by capturing genetic potential that may be masked by environmental variation in phenotype-only selection.

As an illustrative case, we compared three plants from population C_2_ (1–7, 1–12, and 4–3; see Figure 4 and Table 8). Based on the phenotypic D-value alone, plant 4–3 ranked higher than plant 1–7, whereas the exploratory Z-function assigned a higher score to plant 1–7 than to plant 4–3. This difference in ranking may reflect environmental or developmental effects on phenotypic performance. D and Z values were calculated for C_2_ plants with complete phenotypic and marker genotype data, and additional values are provided in Table S4. The three plants shown here are representative examples used only to illustrate that D- and Z-based rankings can differ; they are not the only plants analyzed. However, this descriptive comparison cannot establish that the Z-function better reflects genetic potential or overcomes environmental effects. Validation in larger independent populations and multiple environments is required.

Z = 0.151X_1_Y_1_ + 0.171X_2_Y_2_ − 0.148X_3_Y_3_ + 0.253X_4_Y_4_ + 0.189X_5_Y_5_
 − 0.154X_6_Y_6_− 0.146X_7_Y_7_ + 0.130X_8_Y_8_ (1)

Equation (1). The marker-assisted selection index (Z-function).

Note: Xi (i = 1–8): Standardized phenotypic values for tiller number, stem diameter, kernel number per spike, spike length, spikelet number per spike, grain width, spike width, and plant yield, respectively. Standardization: Xi = (phenotypic value − population mean)/standard deviation. Yi (i = 1–8): Genotypic scores from molecular markers (Y_5_ corresponds to KM33). Scoring: +1 (favorable allele), −1 (unfavorable allele), 0 (heterozygous).

**Table 8:** Information on traits and test results of rye plants.

	Traits	TN (Piece)	SD (cm)	KNS (Piece)	SL (cm)	SNS (Piece)	GW (cm)	SWd (cm)	PY (g)	*D*	*Z*
Samples											
1–7		20	0.35	48.56	8.69	30.67	0.23	0.80	14	0.289	0.383
1–12		29	0.45	66.40	14.52	38.3	0.25	0.94	56	0.714	0.663
4–3		12	0.45	44.57	10.51	30.57	0.21	0.71	12	0.333	−0.399

Note: *D* value is the obtained value of the affiliation function and *Z* value is the obtained value of equation (Equation (1)).

**Figure 4:**
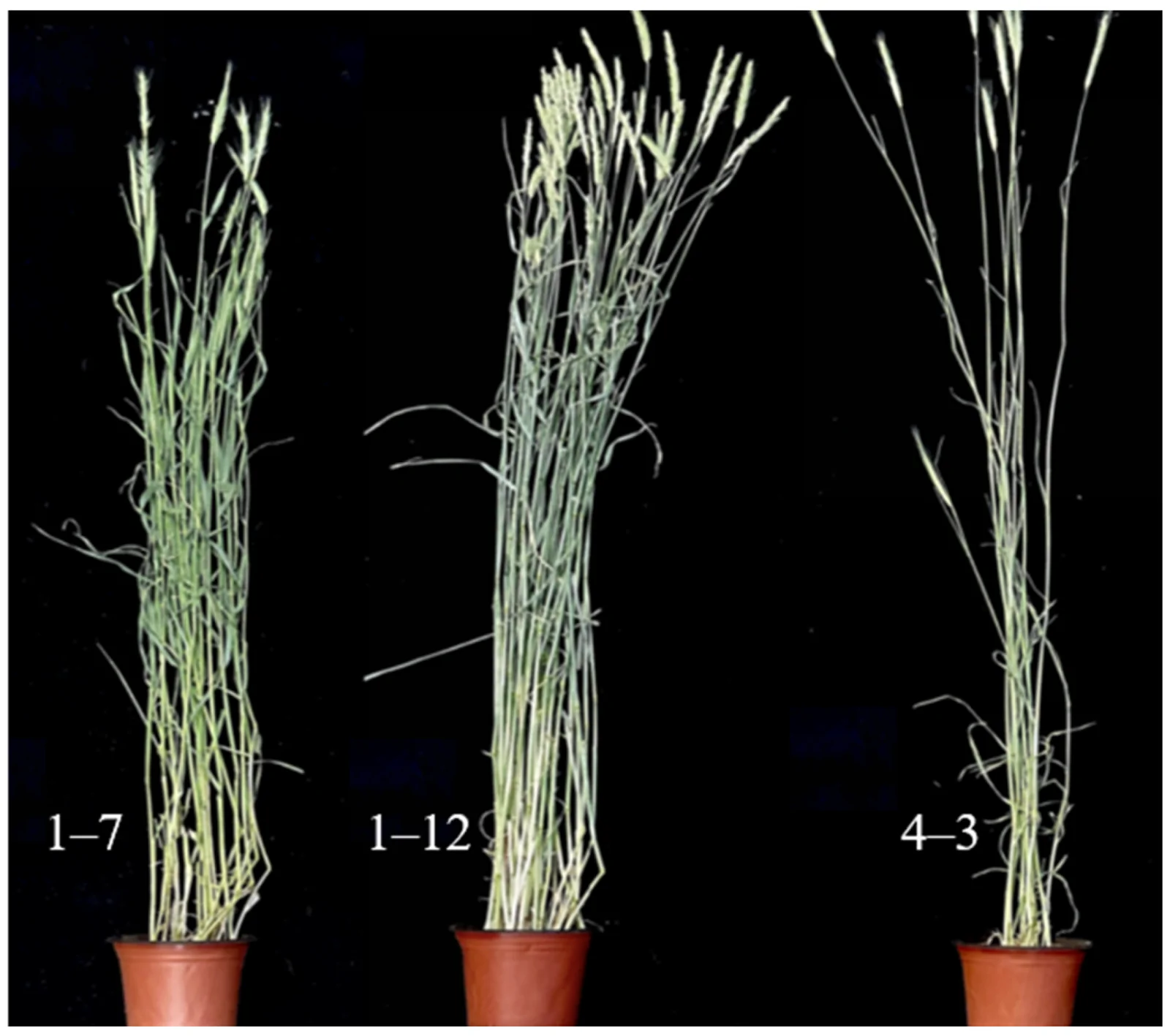
Diagram of three rye plants in C_2_ population.

## 4. Discussion

### 4.1. Gene-Collinearity Analysis near QTL Loci

Because the C_1_ population was phenotyped in a single environment, the estimated QTL effects may be inflated and the mapping resolution limited. Prior to merging, the distribution of the 57 yield-related QTLs originally identified via SLAF-seq [25] was highly uneven across the rye genome. Notably, thirty-eight QTLs were clustered on chromosome 4R, whereas chromosomes 1R, 2R, 3R, and 5R harbored 2–5 QTLs each, and no yield-related QTLs were detected on chromosome 6R. This pattern strongly implicates chromosome 4R as a major hub for the genetic control of yield traits in rye, as genes on this chromosome are mostly involved in regulating plant growth and development. Given the close phylogenetic relationship between rye and wheat, and the established role of the wheat A subgenome in spike development [32], we performed a synteny analysis between the QTL-rich region on rye chromosome 4R and the wheat A subgenome to explore potential functional conservation of yield-related genes. This analysis revealed synteny between the rye 4R QTL hotspot and wheat chromosome 7A, identifying 20 homologous gene pairs (Figure 5). 

Functional annotation of these homologous genes suggested their encoded proteins are predominantly involved in regulating plant growth and development. For example, several encode F-box proteins [33] and zinc finger proteins [34] are both known to function in developmental signaling pathways. Notably, one homologous pair includes the wheat gene *TraesCS7A02G176300.1* (an ERF transcription factor) and its rye counterpart *ScWN4R01G328300.1*. In wheat, this ERF factor enhances grain filling by activating the sucrose transporter gene *TaSUT1*, and its overexpression increases thousand-grain weight and grain width [35]. Another pair comprises the wheat NAC-domain gene *TraesCS7A02G154500.1* and the rye gene *ScWN4R01G359100.1.* NAC transcription factors are known regulators of diverse processes, including phytohormone homeostasis, stress responses, leaf senescence, root development, and seed development/starch accumulation [36]. For instance, overexpression of the wheat NAC gene *TaNAC2-5A* improves root growth, nitrate uptake, and ultimately grain yield [37].

Previous studies on rye genetics have predominantly focused on biotic and abiotic stress tolerance [38,39], with relatively limited attention given to the genetic architecture of yield-related traits [40]. Early QTL mapping studies in rye identified multiple yield-related QTLs and orthologous segments in rice [41,42]. A study performed genome-wide SSR analysis in rye and developed rye-specific markers for detecting rye chromatin in wheat backgrounds [43]. Siekmann et al. [44] reported the presence of yield-related QTL on rye chromosome 4R through GWAS, and further noted that the physical intervals of these QTL were relatively extended. They attributed this observation to suppressed recombination and structural rearrangements, such as inversions and copy number variations, relative to the reference genome. As they discussed, such broad intervals present a substantial challenge for the direct identification of causal genes when relying solely on reference genome coordinates. To complement these findings, the present study employed a high-density genetic map constructed via SLAF-seq. By capitalizing on recombination events within the mapping population rather than depending primarily on a reference assembly, we resolved this chromosomal region into 38 discrete QTLs. This QTL hotspot exhibits strong synteny with wheat chromosome 7A, enabling the identification of candidate genes, including ERF and NAC transcription factors, that are known to regulate grain yield in wheat. To date, most yield-related studies in rye have remained at the stage of QTL mapping, with limited progress in gene cloning and functional characterization [40,45] Collectively, the work of Siekmann et al. [44] established the presence and underlying complexity of yield loci on chromosome 4R, while our study contributes enhanced resolution and candidate-gene resources that may facilitate the eventual deployment of these loci in marker-assisted breeding efforts.

In summary, our findings provide a foundation for developing KASP markers and conducting functional validation of candidate genes, thereby facilitating marker-assisted selection in rye breeding programs. Future work should focus on functional characterization of genes on chromosome 4R to systematically elucidate their roles in determining rye yield.

### 4.2. Analysis of Candidate Genes for Yield-Related Traits

Through functional annotation of genes within the validated QTL intervals and integration of knowledge from orthologous genes in other cereals, we identified several promising candidate genes. These include *ScWN2R01G507400.1*, *ScWN4R01G301500.1*, *ScWN4R01G362100.2*, and *ScWN4R01G352500.1*. These encode a diverse set of proteins, including VQ motif-containing proteins, leucine-rich repeat receptor-like kinases (LRR-RLKs), F-box proteins, CASP-like proteins, zinc finger proteins, plant UDP-glycosyltransferases, and MATH-BTB domain-containing proteins. Substantial evidence from functional studies in cereals confirms the critical roles of these protein families in plant growth and development. 

For example, *ScWN2R01G507400.1* encodes a VQ motif protein, which functions as a transcriptional co-regulator in various signaling pathways. VQ proteins are known to regulate the development of seeds, spikes, flowers, and leaves, and to mediate responses to both biotic and abiotic stresses [46,47]. *ScWN4R01G301500.1* and *ScWN4R01G362100.2* encode LRR-RLKs. LRR-RLKs perceive extracellular signals via ligand binding, leading to intracellular kinase-domain phosphorylation and subsequent signal transduction [48]. This phosphorylation-based mechanism is central to developmental signaling in plants. For instance, the LRR-RLK family in wheat regulates manifold aspects of plant vegetative growth, organ formation, and hormone signaling [49], highlighting their role in reproductive architecture. Similarly, *ScWN4R01G301400.1* and *ScWN4R01G343900.1* encode F-box proteins, components of the ubiquitin-proteasome system that regulate growth, panicle development, and senescence in wheat and rice [33].

Another candidate, *ScWN4R01G335200.1* encodes a UDP-glycosyltransferase. Its rice ortholog, *GSA1* (Grain Size and Abiotic stress tolerance 1/*OsUGT83A1*), a typical UDP-glycosyltransferase, positively regulates grain size, as overexpression significantly impacts grain dimensions [50]. *ScWN4R01G335100.1* encodes a zinc finger protein, a class of regulators involved in growth and development [51], flowering time [52], and intracellular signaling [53]. Finally, *ScWN4R01G352500.1* encodes a MATH-BTB domain containing protein, belonging to the BTB protein superfamily. MATH-BTB proteins are implicated in maintaining cellular homeostasis and regulating organ development [54]. For example, in rice, they function in organ development [55], while in *Arabidopsis*, the stability of the BPM1 protein is regulated by environmental cues, thereby influencing flowering time and abiotic stress responses [56].

Collectively, the functional convergence of these candidate genes on growth and developmental regulation highlights the complexity of yield-related genetic architecture on rye chromosome 4R and provides a foundation for their functional validation. Nevertheless, it should be emphasized that the evidence supporting these candidate genes is currently based on positional information and expression profiles in related species, not on direct functional studies in rye. These genes therefore represent candidate genes requiring further functional characterization rather than confirmed causal genes. Related work will be carried out in future studies to elucidate their specific roles in rye yield formation.

### 4.3. Exploratory Evaluation of the Marker-Assisted Selection Index

Advances in low-cost, high-throughput gene sequencing have diversified the methodologies available for crop breeding [57]. Approaches such as genome-wide association studies (GWASs) and genomic prediction are now integral to modern breeding programs. Successful GP models in wheat, maize, and soybean, for example, have proven valuable for guiding selection decisions [58]. Similarly, trait-specific gene-assisted selection models have enhanced breeding efficiency in crops like apple [59].

In this study, we integrated QTL effect estimates (PVE), molecular marker genotypes, and phenotypic data to construct a marker-assisted selection index (Z-function). Multiple marker–trait comparisons were performed without multiple-testing correction, in-creasing the risk of false-positive associations. Some genotype classes were small, and the C_2_ field experiment was not a replicated randomized trial. Therefore, the marker–trait associations should be considered preliminary, and the Z-function remains an exploratory index rather than a validated predictor of genetic potential. Given that self-incompatibility in rye poses a core obstacle to genetic research and breeding improvement, the development of such an index represents a practical effort to facilitate selection in this cross-pollinated species. The PVE coefficients were derived from the single-environment C_1_ QTL analysis, and their stability across environments has not been established. In addition, C_1_ (♀Z1672 × ♂89R41) and C_2_ (♀90R13 × ♂Z837) are different crosses, and the C_2_ marker–trait associations should therefore be interpreted as evidence of marker informativeness in an independent genetic background rather than as validation of the C_1_ QTL effects. As more genotypic and phenotypic data from diverse rye germplasm become available, the weighting parameters of this index can be further refined. Future research should therefore prioritize independent validation of the index in larger populations, replicated field trials, and multi-environment experiments, while also applying appropriate multiple-testing correction and using shared markers to establish genetic correspondence between populations. Until such validation is available, the Z-function should be regarded as an exploratory index rather than a validated predictor of genetic potential or a ready-to-use selection tool.

## 5. Conclusions

Our previous SLAF-seq analysis [25] in population C_1_ identified 57 QTLs for yield-related traits, which clustered into 28 genomic regions, suggesting a polygenic architecture with possible pleiotropic or tightly linked effects. Because self-incompatibility in *S*. subsp. *segetale* prevents the development of inbred lines, the double pseudo-testcross F_1_ design was essential for QTL detection. However, it also limits formal QTL validation through conventional segregating populations. To assess marker utility across genetic backgrounds, we developed closely linked SNP and SSR markers from these QTLs and screened them in diverse rye populations (C_2_, A_3_–A_7_), retaining eight SSR and fifteen KASP markers with reliable polymorphism. Although cross-population informativeness does not constitute formal QTL validation, it indicates that these marker loci may retain diagnostic value in different crosses. With the current data, the genetic relationships between alleles in the two populations cannot be fully resolved. Larger populations, shared markers across multiple crosses, and multi-environment trials will be needed to determine whether the same causal variants or linked loci are involved. Therefore, this study should be regarded as a preliminary exploration of the potential utility of these markers across populations. For the genomic intervals covered by these markers, we prioritized nine candidate genes based on positional evidence and functional conservation with known yield-related genes in Poaceae. We constructed a preliminary marker-assisted selection index integrating QTL PVE values, marker genotypes, and standardized phenotypes. This index should be regarded as an exploratory selection tool whose predictive ability requires testing in larger independent populations and multi-environment trials. Collectively, the distribution of QTLs across multiple linkage groups and their clustering into 28 regions support a quantitative, polygenic basis for yield in rye, consistent with findings in other cereals, and highlight genomic regions that may simultaneously affect multiple traits through pleiotropy or tight linkage.

## Figures and Tables

**Figure 1:**
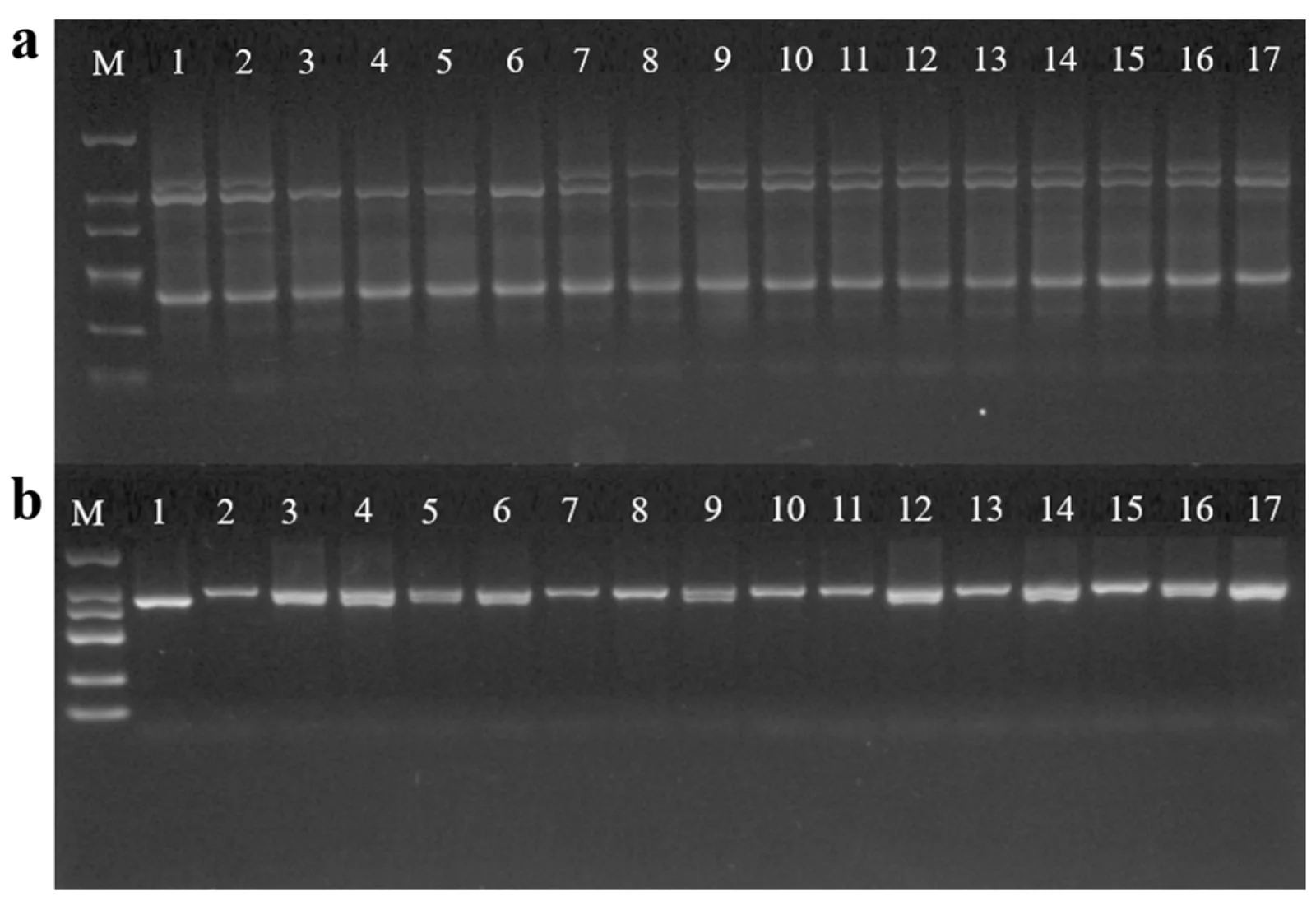
Partial results of SSR18 and SSR26 in C_2_ population verification. (**a**) Upper panel, electrophoresis gel image for spike width genotyping using SSR18. Lane M, 2000 bp DNA marker (bands from top to bottom: 2 kb, 1 kb, 750 bp, 500 bp, 250 bp, 100 bp); lane 1, heterozygote; lane 3, allele 1; lane 8, allele 2. All lanes are plants from the C_2_ population, with genotypes as indicated. (**b**) Lower panel: Electrophoresis gel image for single-plant yield genotyping using SSR26. Lane M, 2000 bp DNA marker as in (**a**); lane 1, allele 1; lane 2, allele 2; lane 3, heterozygote. All lanes are plants from the C_2_ population, with genotypes as indicated.

**Figure 2:**
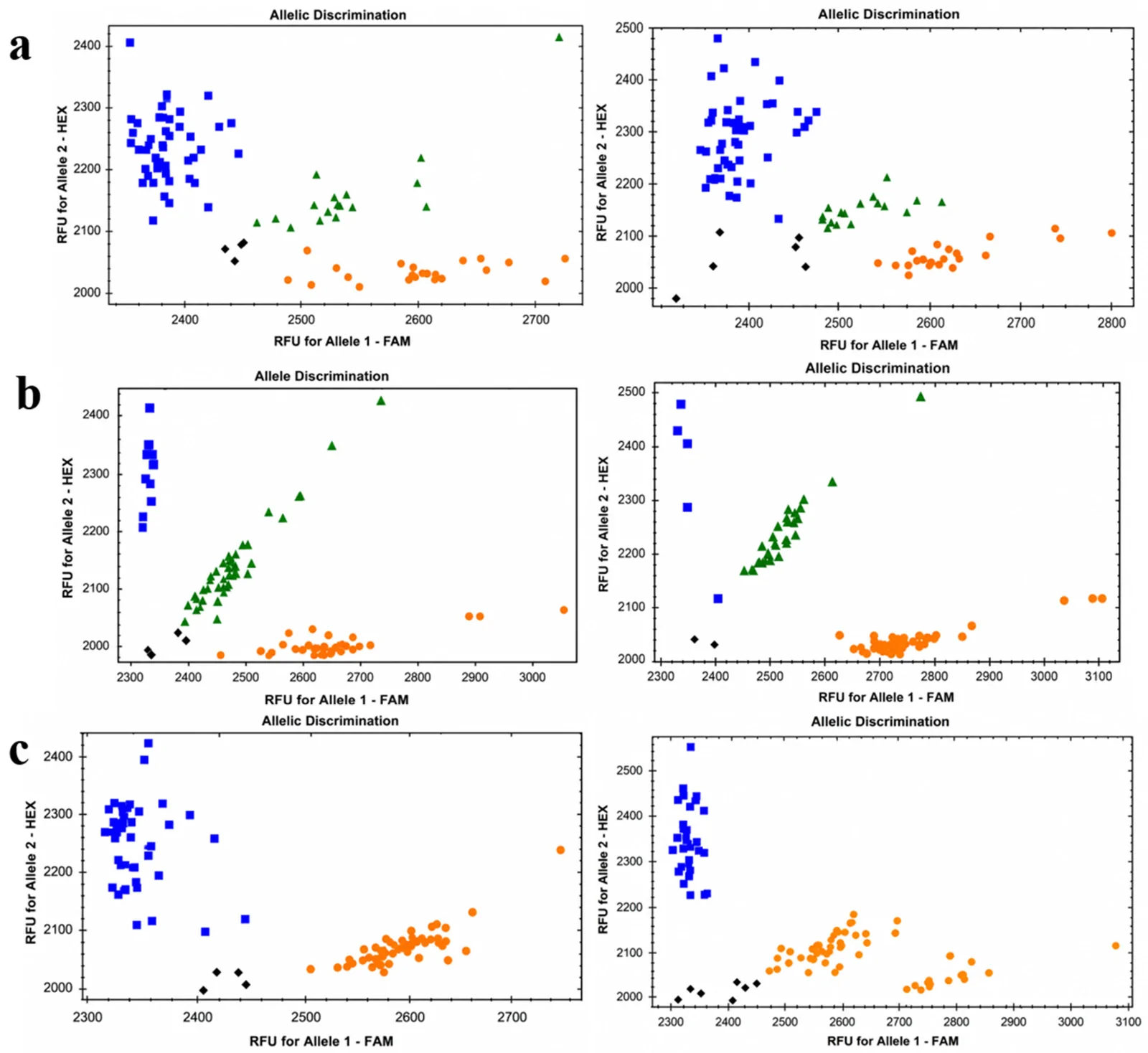
Genotyping plot of selected KASP markers in C_2_ population. (**a**) KM26, (**b**) KM33, (**c**) KM42. The plot displays fluorescence signals: the *X*-axis represents Allele 1 (FAM dye), and the *Y*-axis represents Allele 2 (HEX dye). Blue, homozygous for allele 1; orange, homozygobus for allele 2; green, heterozygous; black, undetermined genotype (no call).

**Figure 3:**
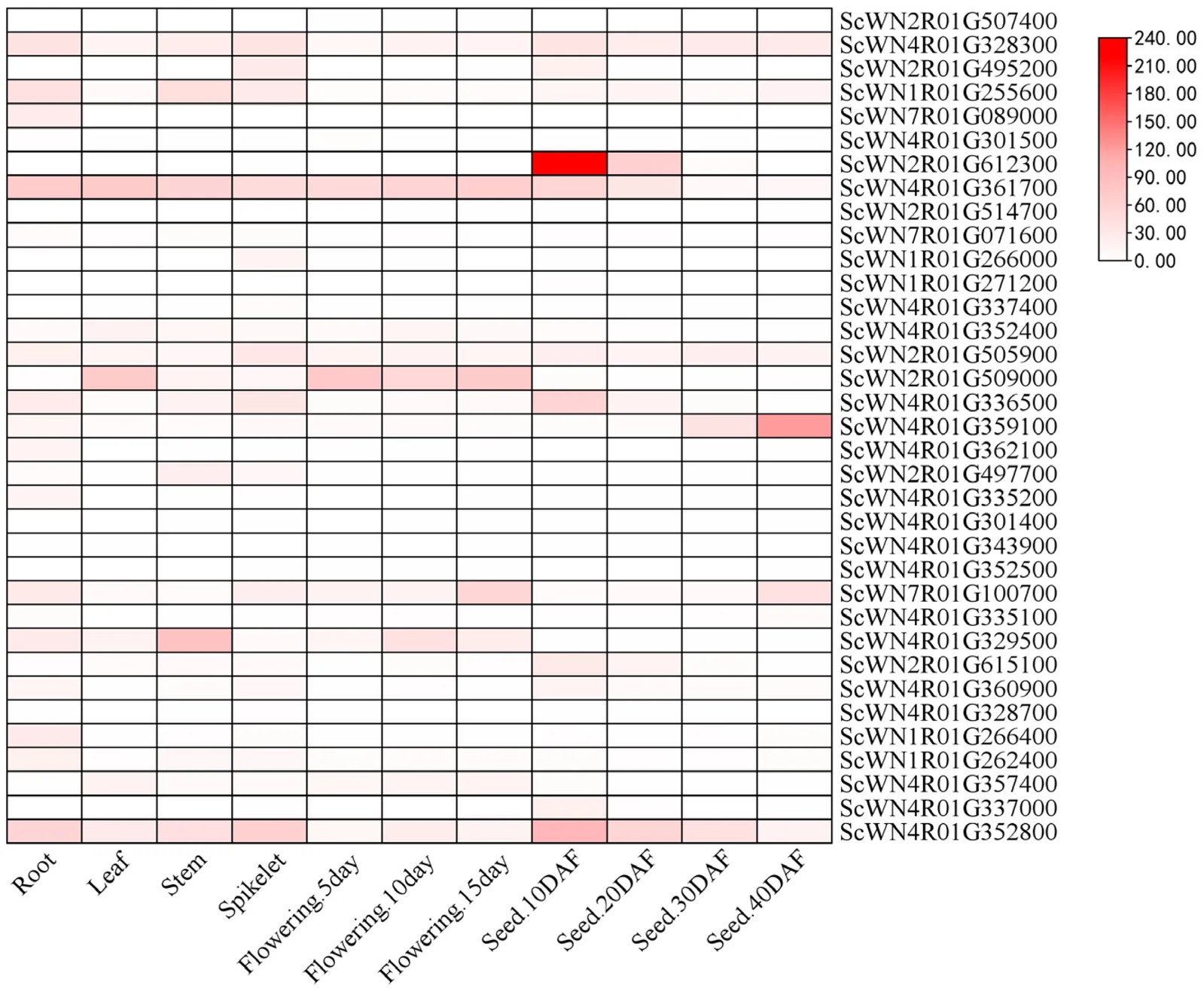
Heatmap of expression patterns for the 35 annotated candidate genes across different tissues. Tissue abbreviations: flowering. Five day, ten day, fifteen day: flowers at five, ten, and fifteen days after flowering; Seed. 10 DAF, 20 DAF, 30 DAF, 40 DAF: grains at 10, 20, 30, and 40 DAF.

**Figure 5:**
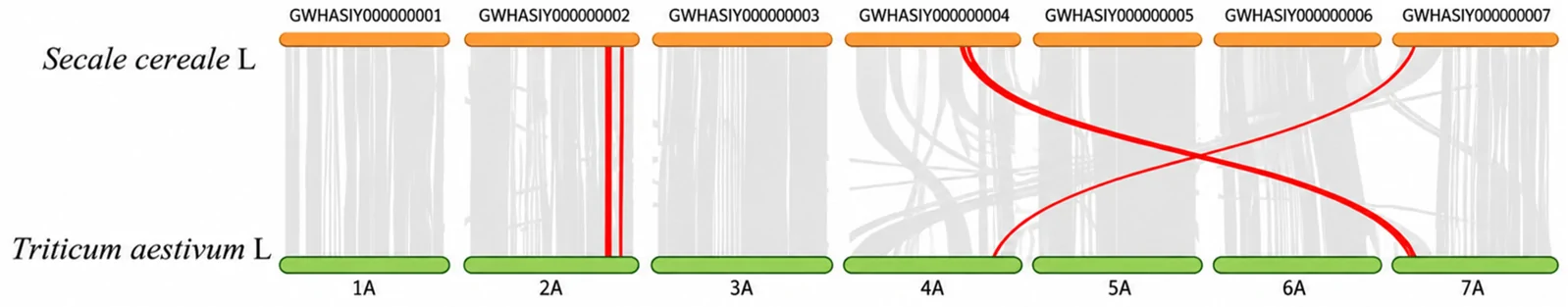
Rye and wheat A genome covariance map.

**Table 1:** Rye population used in this study.

Population Number	Population Source
C_1_	(♀) Z1672 × (♂) 89R41
C_2_	(♀) 90R13 × (♂) Z837
A_3_	89R39
A_4_	89R42
A_5_	89R58
A_6_	90R33
A_7_	89R44

Note: R stands for *S. cereale* subsp. *segetale* and Z stands for cultivated rye.

**Table 2:** Summary of candidate QTLs for yield-related traits in rye.

Trait	QTL Name	Chromosome	Peak Position (cM)	1-LOD Support Interval (cM)	Peak LOD	PVE at Peak (%)	No. of Supporting SNPs
TN	*qTN-5*	Chr.5	155.138	155.138–155.198	3.057	13.13	5
	*qTN-7*	Chr.7	18.648	/	3.558	15.11	7
ATN	*qATN-7*	Chr.7	18.648	/	3.558	13.64	7
PH	*qPH-4*	Chr.4	68.533	/	3.043	13.08	3
ESL	*qESL-7*	Chr.7	18.011	/	4.763	19.70	3
SD	*qSD-2*	Chr.2	110.676	110.676–110.994	4.197	17.57	4
BY	*qBY-2*	Chr.2	110.676	/	2.565	11.14	4
	*qBY-4*	Chr.4	116.298	116.093–116.298	2.618	11.36	4
SL	*qSL-4*	Chr.4	73.882	/	6.332	25.29	3
SWd	*qSWd-4a*	Chr.4	64.028	60.609–65.893	3.701	15.67	2
	*qSWd-4b*	Chr.4	73.882	/	3.8	16.05	3
	*qSWd-4c*	Chr.4	77.871	77.871–83.463	3.881	16.37	27
	*qSWd-5*	Chr.5	103.006	/	3.441	14.65	8
RL	*qRL-4*	Chr.4	152.886	/	4.692	19.43	4
SNS	*qSNS-2*	Chr.2	148.73	/	4.547	18.89	10
KNS	*qKNS-4a*	Chr.4	65.893	64.028–65.893	3.542	15.05	8
	*qKNS-4b*	Chr.4	73.882	/	3.114	13.36	3
KWS	*qKWS-4*	Chr.4	64.774	64.028–65.893	4.469	18.6	2
SWg	*qSWg-4a*	Chr.4	65.893	65.520–65.893	4.272	17.86	8
	*qSWg-4b*	Chr.4	73.882	/	4.301	17.97	3
GL	*qGL-4*	Chr.4	73.882	/	4.621	19.17	3
GW	*qGW-4*	Chr.4	77.871	77.871–81.264	3.635	15.41	27
	*qGW-7*	Chr.7	38.138	/	3.831	16.17	19
TGW	*qTGW-3*	Chr.3	158.144	158.144–160.838	3.396	14.48	3
	*qTGW-5*	Chr.5	99.844	/	4.344	18.13	3
PY	*qPY-1a*	Chr.1	9.583	/	3.024	13	4
	*qPY-1b*	Chr.1	39.252	/	3.031	13.03	14
	*qPY-7*	Chr.7	30.522	/	3.277	14.01	3

Note: QTL names with suffixes ‘a’, ‘b’, ‘c’ indicate multiple QTL clusters detected on the same chromosome for the same trait, separated by genetic distance gaps (>5 cM). The 1-LOD support interval was assigned when multiple significant markers were clustered within a continuous genetic distance; otherwise, the peak position is presented as a single point. Peak positions were selected based on the highest LOD score within each cluster; when LOD scores were tied, the position with the greater number of supporting SNPs was chosen. PVE values represent the explained phenotypic variance at the peak marker only, and are not cumulative across markers within the same interval. The number of supporting SNPs indicates the count of SNP markers co-segregating at the peak position.

**Table 3:** Characteristics of the eight validated SSR markers.

Trait	Markers	Primer		Length (bp)
ATN	SSR2	F	GCTCTTGGAACGGATTTG	1651
		R	GCAGCCTCTTCGGACAT	
SWd	SSR8	F	CACAAACACCGCAAAGAACCACG	171
		R	CGCCGATGCTGTTGCCCATT	
	SSR10	F	TCTATCTGAGTGCCCTGAC	388
		R	TTATTGCGGCTTACCTT	
	SSR18	F	CTGTCCACCCACATACCA	1227
		R	TTACTTCACGTCGGCAAT	
PY	SSR26	F	CCTTGTCTCCTGTGGCTTTA	945
		R	CATCGCATTTCCAGTTCC	
RL	SSR34	F	ATCGGTGAATGCTGCTCG	252
		R	TGGACCCATCGCCAAAGT	
TGW	SSR51	F	CTACGCACCGAAAGTGG	1285
		R	GGCTGCCTGCATACGA	
BY	SSR52	F	CGGGTATAATGCGAATGG	809
		R	GGAAGTAGATGCCGAACAA	

**Table 4:** Association analysis between SSR marker genotypes and phenotypic traits.

Markers	Traits	Allele 1		Allele 2		Heterozygote		*p*
		*n*	Mean Value	*n*	Mean Value	*n*	Mean Value	
SSR18	SWd (cm)	134	0.7674	10	0.8270	158	0.7623	0.011
SSR26	PY (kg)	156	0.0453	36	0.0359	148	0.0419	0.037

Note: Total *n* = 376 (C_2_ population). Missing counts per marker indicate amplification failures.

**Table 5:** Primer sequences and target SNPs for the 15 validated KASP assays.

Markers	Primer	SNP
KM1	GAAGGTGACCAAGTTCATGCTcgttcatagatgtgctatcgaacA	A/T
	GAAGGTCGGAGTCAACGGATTcgttcatagatgtgctatcgaacT	
	tgtacacgtgcaccatagca	
KM3	GAAGGTGACCAAGTTCATGCTgtagctggagtcgaggtagtC	C/T
	GAAGGTCGGAGTCAACGGATTgtagctggagtcgaggtagtT	
	agtctgacaaactcggcagg	
KM7	GAAGGTGACCAAGTTCATGCTcaccactaccttagtctctcttgG	G/T
	GAAGGTCGGAGTCAACGGATTcaccactaccttagtctctcttgT	
	tcgttgtgtgagtggaggtg	
KM10	GAAGGTGACCAAGTTCATGCTcttggtactaaagacaatgttgcA	A/G
	GAAGGTCGGAGTCAACGGATTcttggtactaaagacaatgttgcG	
	ggtccattacatgtatccctacca	
KM11	GAAGGTGACCAAGTTCATGCTttgacttcaccacttccggG	G/A
	GAAGGTCGGAGTCAACGGATTttgacttcaccacttccggA	
	gctatgccagcgaccaaaag	
KM15	GAAGGTGACCAAGTTCATGCTccaatcccaaacatacgtcactG	G/C
	GAAGGTCGGAGTCAACGGATTccaatcccaaacatacgtcactC	
	tgtgcttgtccactgccaat	
KM18	GAAGGTGACCAAGTTCATGCTccgatcagtgaccggcgA	A/G
	GAAGGTCGGAGTCAACGGATTccgatcagtgaccggcgG	
	acatcccaaaccggccttaa	
KM23	GAAGGTGACCAAGTTCATGCTaggttatcgtccttgactgtgaG	G/A
	GAAGGTCGGAGTCAACGGATTaggttatcgtccttgactgtgaA	
	ggcataccctcaatgacggt	
KM24	GAAGGTGACCAAGTTCATGCTcaagccaaaccggaacaagT	T/C
	GAAGGTCGGAGTCAACGGATTcaagccaaaccggaacaagC	
	cccgtcattgtggaagacca	
KM25	GAAGGTGACCAAGTTCATGCTagtgatcctgtcaaaatcgtcA	A/G
	GAAGGTCGGAGTCAACGGATTagtgatcctgtcaaaatcgtcG	
	cagaggcttcgggaaagtga	
KM26	GAAGGTGACCAAGTTCATGCTtggtttgggccactagatcG	G/A
	GAAGGTCGGAGTCAACGGATTtggtttgggccactagatcA	
	tcgggattgctagagcttcg	
KM33	GAAGGTGACCAAGTTCATGCTccactggcatggtactgtagtC	C/T
	GAAGGTCGGAGTCAACGGATTccactggcatggtactgtagtT	
	ggatggcgatgacaatgcag	
KM35	GAAGGTGACCAAGTTCATGCTcccagcctccaagagaagaC	C/T
	GAAGGTCGGAGTCAACGGATTcccagcctccaagagaagaT	
	acaaacgggttcacctcttca	
KM36	GAAGGTGACCAAGTTCATGCTccatttttctggaagcacaactG	G/T
	GAAGGTCGGAGTCAACGGATTccatttttctggaagcacaactT	
	cttccacgagatgcacacct	
KM42	GAAGGTGACCAAGTTCATGCTtgatagaggagtcgtcgacC	C/T
	GAAGGTCGGAGTCAACGGATTtgatagaggagtcgtcgacT	
	cttgatggtgacctcggagg	

**Table 6:** Association analysis between KASP marker genotypes and phenotypic traits.

Markers	Traits	Allele 1		Allele 2		Heterozygote		*p*
		*n*	Mean Value	*n*	Mean Value	*n*	Mean Value	
KM1	TN (piece)	36	28.97	258	38.37	0	0.00	9.25 × 10^−4^
KM7	SD (cm)	79	0.4456	76	0.4289	133	0.4425	7.00 × 10^−3^
KM26	SL (cm)	138	13.87	81	12.99	41	13.72	8.50 × 10^−5^
KM33	SNS (piece)	29	33.04	132	36.04	132	34.97	2.00 × 10^−3^
KM36		98	34.53	67	36.28	49	34.95	9.00 × 10^−3^
KM42	GW (cm)	122	0.2319	147	0.2265	4	0.2125	1.80 × 10^−2^
KM10	KNS (piece)	70	56.68	101	48.14	35	55.09	4.00 × 10^−6^

Note: Total *n* = 376 (C_2_ population). Missing counts per marker indicate undetermined genotypes (no call).

**Table 7:** Functional annotation of candidate genes associated with yield-related traits in rye.

Traits	Gene	PFAMs
TN	*ScWN7R01G089000.1*	Sugar_tr
	*ScWN7R01G100700.1*	MatE
	*ScWN7R01G071600.1*	DnaJ, Jiv90
SD	*ScWN2R01G505900.1*	CaATP_NAI, Cation_ATPase, Cation_ATPase_C, Cation_ATPase_N, E1-E2_ATPase, Hydrolase
	*ScWN2R01G497700.1*	p450
	*ScWN2R01G507400.1*	VQ
	*ScWN2R01G495200.1*	ADH_N, ADH_zinc_N
	*ScWN2R01G509000.1*	PAP_fibrillin
	*ScWN2R01G514700.1*	p450
KNS	*ScWN4R01G301500.1*	LRRNT_2, LRR_1, LRR_8
	*ScWN4R01G301400.1*	F-box
SL	*ScWN4R01G329500.1*	K-box, SRF-TF
	*ScWN4R01G328700.2*	SKIP_SNW
SNS	*ScWN2R01G612300.1*	LTP_2
	*ScWN2R01G615100.1*	CASP family
GW	*ScWN4R01G337000.1*	F-box, START
	*ScWN4R01G352800.1*	CHDCT2, Chromo, DUF1086, DUF1087, Helicase_C, PHD, SNF2_N
	*ScWN4R01G335100.1*	zf-RING_2, zinc_ribbon_9
	*ScWN4R01G335200.1*	UDPGT
	*ScWN4R01G343900.1*	F-box, FBD
	*ScWN4R01G352400.1*	Myb_DNA-binding, Response_reg
	*ScWN4R01G328300.1*	GRAM
	*ScWN4R01G336500.1*	F-box
	*ScWN4R01G337400.1*	F-box, START
	*ScWN4R01G352500.1*	BTB, MATH
SWd	*ScWN4R01G361700.1*	Peptidase_S10
	*ScWN4R01G360900.1*	RLK
	*ScWN4R01G359100.1*	NAC
	*ScWN4R01G362100.2*	LRRNT_2, LRR_1, LRR_4, LRR_6, LRR_8, Pkinase, Pkinase_Tyr
	*ScWN4R01G357400.1*	Calreticulin, DYW_deaminase, Jacalin, PPR, PPR_2, PPR_3
PY	*ScWN1R01G266000.1*	Inhibitor_I29, Peptidase_C1
	*ScWN1R01G262400.1*	IBR
	*ScWN1R01G271200.1*	PP2C
	*ScWN1R01G255600.1*	PTR2
	*ScWN1R01G266400.1*	LysM

## Data Availability

The SSR and KASP marker sequences developed in this study were included in this paper. The data of wheat A genome and rye (Weining) genome were obtained from the NGDC database (https://ngdc.cncb.ac.cn/). Transcript expression profiles of rye genes in different tissues were obtained from the WheatOmics database (http://wheatomics.sdau.edu.cn/). All other relevant data supporting the conclusions of this article are included within the article and its additional files.

## Supplementary Materials

The following supporting information can be downloaded at: https://www.mdpi.com/article/10.3390/genes17101179/s1, Table S1: The PCR reaction mixture and amplification program. Table S2: KASP PCR reaction system, composition of the KASP primer mixture and thermocycling program for KASP assays. Table S3: Details of candidate QTLs for yield-related traits in rye. Table S4: Detailed derivation of the Z-Function. D and Z values of other individual plants. Table S5: Marker–trait correspondence. Table S6: *p*-values for each trait under SSR18 genotyping. Figure S1 Normality test results, histogram, and normal Q–Q plot for tiller number (TN). Figure S2 Normality test results, histogram, and normal Q–Q plot for kernel number per spike (KNS).

## Abbreviations

The following abbreviations are used in this manuscript:

ATN	available tiller number
BY	biological yield
cM	centiMorgan
CP	cross-pollinated
ESL	ear of grain-stem node length
GAP	genomics-assisted prediction
GL	grain length
GW	grain width
KNS	kernel number per spike
KWS	kernel weight per spike
LOD	logarithm of odds
PH	plant height
PVE	phenotypic variation explained
PY	plant yield
QTLs	quantitative-trait loci
RL	root length
SD	stem diameter
SL	spike length
SLAF-seq	specific-locus amplified-fragment sequencing
SNPs	single nucleotide polymorphisms
SNS	spikelet number per spike
SSRs	simple sequence repeats
SW	spike weight
SWd	spike width
TGW	thousand-grain weight
TN	tiller number
