# Supplementary material for: Development of Molecular Markers and a Preliminary Selection Index for Yield-Related QTLs in Secale cereale subsp. segetale
Source: Genes (Basel). 2026 Sep 24;17(10):1179. doi: 10.3390/genes17101179 (PMC13704579; doi:10.3390/genes17101179)
Supplement: Supplementary file 1 [file genes-17-01179-s001.zip › Additional file 2.pdf]

Additional file 2. Visualization of a subset of the phenotypic data

Tests of Normality

	Kolmogorov-Smirnov[a]			Shapiro-Wilk		
	Statistic	df	Sig.	Statistic	df	Sig.
TN	.043	268	.200*	.992	268	.131

*. This is a lower bound of the true significance.

a. Lilliefors Significance Correction

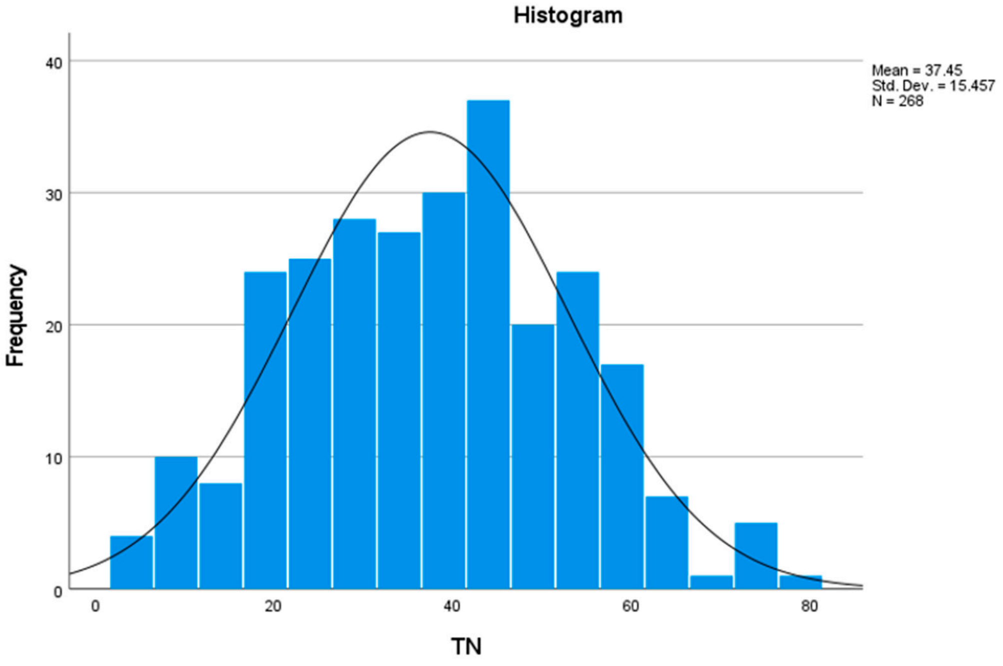

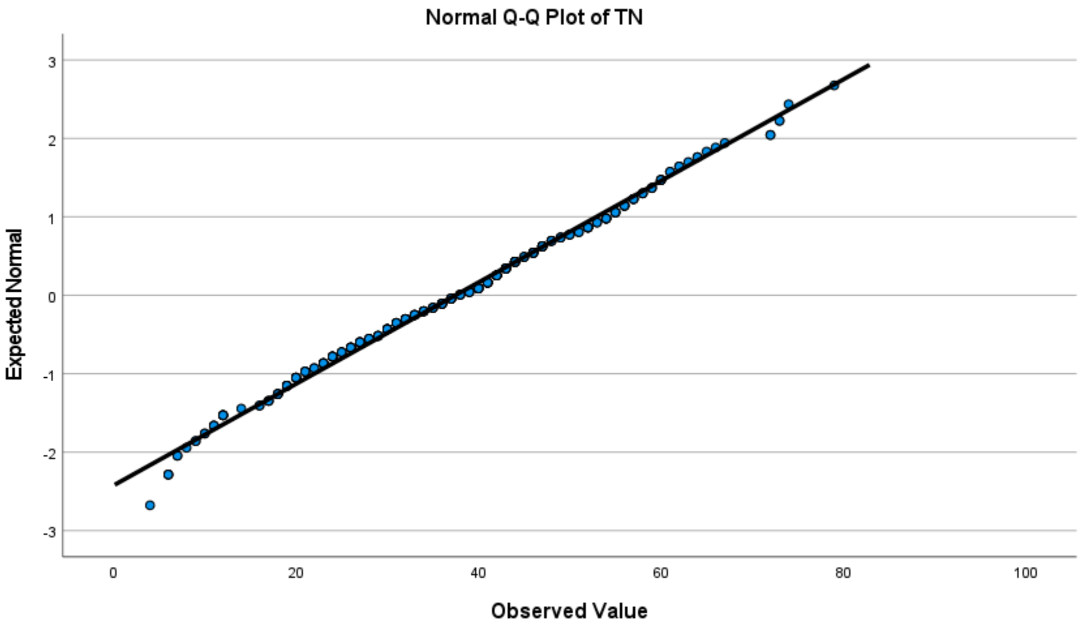

Figure S1 Normality test results, histogram, and normal Q-Q plot for tiller number (TN).

Tests of Normality

	Kolmogorov-Smirnov[a]			Shapiro-Wilk		
	Statistic	df	Sig.	Statistic	df	Sig.
KNS	.055	206	.200*	.990	206	.164

*. This is a lower bound of the true significance.

a. Lilliefors Significance Correction

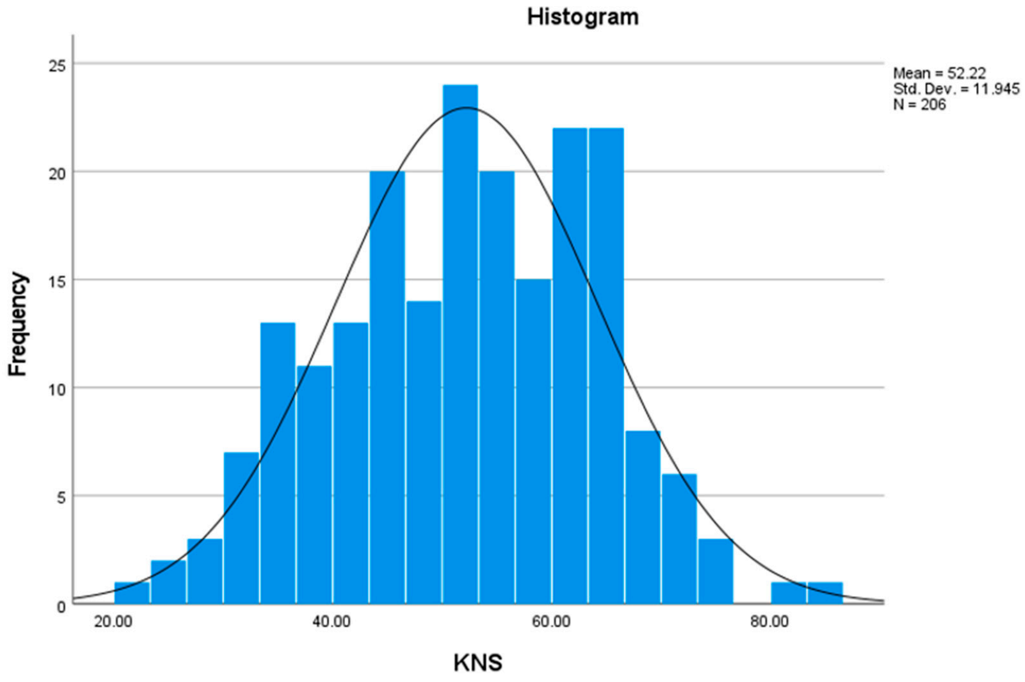

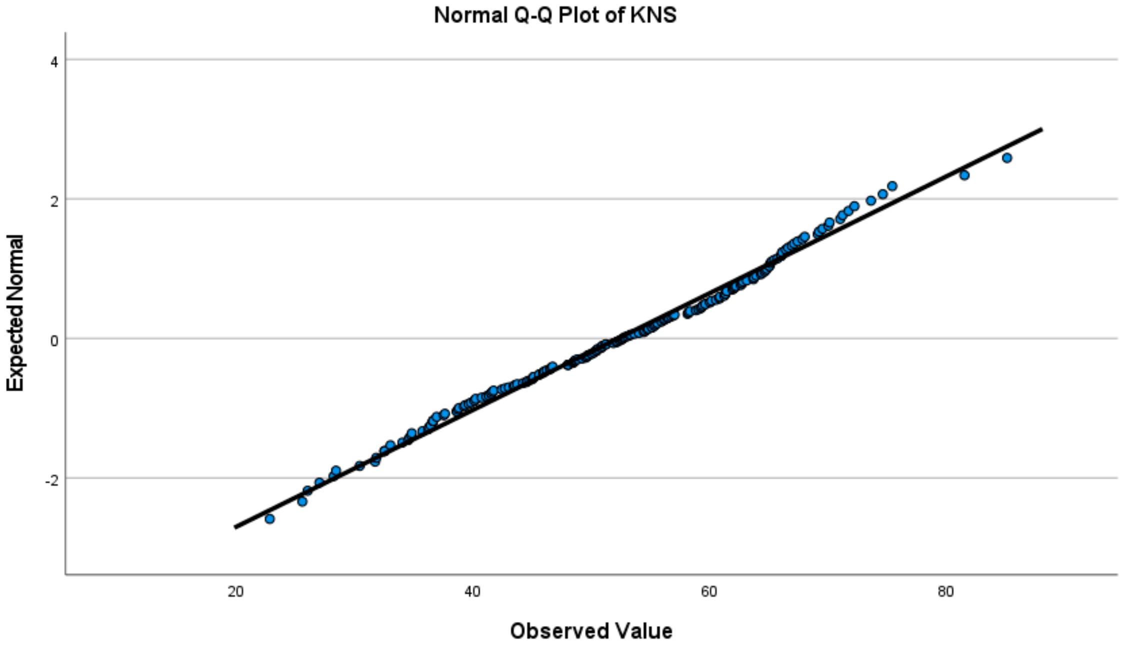

Figure S2 Normality test results, histogram, and normal Q-Q plot for kernel number per spike (KNS).
